# Head-to-Head Comparison of Response Rates to the Two mRNA SARS-CοV-2 Vaccines in a Large Cohort of Solid Organ Transplant (SOT) Recipients

**DOI:** 10.3390/vaccines10020190

**Published:** 2022-01-25

**Authors:** Smaragdi Marinaki, Dimitrios Degiannis, Sotirios Roussos, Efstathios Xagas, Paraskevi Tsoutsoura, Stamatis Adamopoulos, Vana Sypsa, Antigoni Chaidaroglou, Ioanna D. Pavlopoulou, Angelos Hatzakis, Ioannis N. Boletis

**Affiliations:** 1 Clinic of Nephrology and Renal Transplantation, Laiko General Hospital, Medical School of Athens, National and Kapodistrian University, 11527 Athens, Greece; exagas@med.uoa.gr (E.X.); laikneph@laiko.gr (P.T.); inboletis@med.uoa.gr (I.N.B.); 2 Molecular Immunopathology and Histocompatibility Unit, Onassis Cardiac Surgery Center, 17674 Athens, Greece; degianis@ocsc.gr (D.D.); chaidaroglou@ocsc.gr (A.C.); 3 Department of Hygiene, Epidemiology & Medical Statistics, School of Medicine, National and Kapodistrian University of Athens, 15772 Athens, Greece; sotirisr@med.uoa.gr (S.R.); vsipsa@med.uoa.gr (V.S.); ahatzak@med.uoa.gr (A.H.); 4 Heart Failure and Transplant Units, Onassis Cardiac Surgery Center, 17674 Athens, Greece; stamatis.adamo@gmail.com; 5 Pediatric Research Laboratory, Faculty of Nursing, National and Kapodistrian University of Athens, 15772 Athens, Greece; idpavlop@nurs.uoa.gr

**Keywords:** SARS-CοV-2 vaccination, clinical transplantation, mRNA vaccines, solid organ transplant recipients

## Abstract

Due to their higher risk of developing life-threatening COVID-19 disease, solid organ transplant (SOT) recipients have been prioritized in the vaccination programs of many countries. However, there is increasing evidence of reduced immunogenicity to SARS-CοV-2 vaccination. The present study investigated humoral response, safety, and effectiveness after the two mRNA vaccines in 455 SOT recipients. Overall, the antibody response rate was low, at 39.6%. Higher immunogenicity was detected among individuals vaccinated with the mRNA1273 compared to those with the BNT162b2 vaccine (47% vs. 36%, respectively, *p* = 0.025) as well as higher median antibody levels of 31 (7, 372) (AU/mL) vs. 11 (7, 215) AU/mL, respectively. Among the covariates assessed, vaccination with the BNT162b2 vaccine, antimetabolite- and steroid-containing immunosuppression, female gender, the type of transplanted organ and older age were factors that negatively influenced immune response. Only mild adverse effects were observed. Our findings confirm poor immunogenicity after vaccination, implicating a reevaluation of vaccination policy in SOT recipients.

## 1. Introduction

Solid organ transplant (SOT) recipients represent a high-risk group for all severe acute respiratory syndrome coronavirus 2 (SARS-CoV-2) infection-related adverse outcomes, including mortality [1]. Τhe incidence of COVID-19 among kidney transplant patients is not particularly high, but when they get infected, this is related to significant morbidity and mortality. Infection rates range from 0.27% to 1.67%, but they depend greatly on the number of tested individuals. The presence of at least one comorbidity is an almost universal finding in transplanted patients. During the first and the second wave of the pandemic, case fatality rates (CFR) at approximately 20% had been reported. Compared to the outcomes of influenza in solid organ transplant (SOT) recipients, all adverse outcomes were higher in those with COVID-19 infection: 93% vs. 70% hospitalization rate and 30% vs. 16% ICU admission, respectively [2]. However, SOT recipients have been excluded from all phase three vaccination trials [3].

After their prioritization for coronavirus disease 2019 (COVID-19) vaccination in most countries, there is increasing evidence about alarmingly low response rates to both mRNA vaccines. In Greece, vaccination of SOT recipients began in January and until March, all SOT recipients had access to a two-dose vaccination. In a recently published systematic review, the pooled estimate of antibody response after vaccination in SOT recipients was 35% [4].

This finding, though disappointing, is not surprising: besides a suboptimal immune response to viral pathogens, immunocompromised status is associated with reduced immunogenicity to vaccination, as known from overall response rates to other vaccines [5,6].

Notably, the humoral response of SOT recipients to SARS-CοV-2 vaccines is inferior to other immunocompromised patient groups. In a recent study by Haidar et al., among different immunosuppressive conditions, seropositivity was lowest in SOT recipients; 37.2% compared to 54.7% and 82.4% in patients with hematologic malignancies and solid tumors, respectively. In the same study, response rates of HIV patients were 94% [7].

Most disturbingly, there are reports describing breakthrough COVID-19 infections among fully vaccinated SOT recipients with variable and often severe disease courses, implicating an urgent need for scientific evidence that could lead to a reevaluation of vaccination policies among the above group of patients [8].

The present study assessed humoral response, safety, and clinical effectiveness after full vaccination with one of the two mRNA SARS-CοV-2 vaccines in a large cohort of 455 SOT recipients. Furthermore, the difference in response rates between the BNT162b2 (Pfizer/BioNTech) and the mRNA1273 (Moderna) vaccine was investigated.

## 2. Materials and Methods

We included a total of 455 consecutive SOT recipients vaccinated with either of the two authorized mRNA SARS-CοV-2 vaccines. All SOT recipients had completed vaccination with either the BNT162b2 or mRNA1273 vaccine and were thereafter invited by telephone to participate in the study. Individuals agreeing to participate were scheduled for a blood specimen three to four weeks after the second vaccine dose in order to determine their humoral response. After signing a written informed consent form, participants filled out a questionnaire including demographics, previous COVID-19 infection, and early post-vaccination side-effects after each vaccine dose. Previous COVID-19 infection was not an exclusion criterion since, according to our vaccination policy, all patients recovering after natural infection were advised to proceed to a full vaccination schedule two months later.

Clinical data on transplantation status, immunosuppression, comorbidities, and concomitant medications were obtained from medical charts. All laboratory tests and levels of immunosuppressive medications were performed in the central laboratory of the two referral hospitals and were subsequently recorded in the patients’ charts. The estimated glomerular filtration rate (eGFR) was calculated using the Chronic Kidney Disease Epidemiology Collaboration (CKD-EPI) equation.

Antibodies were measured at a median of 28 days after the second vaccine dose (range, 22 to 33 days) utilizing a chemiluminescent microparticle immune assay (CMIA) which quantifies IgG antibodies against the receptor-binding domain (RBD) of the S1 subunit of the spike protein of SARS-CoV-2 (Abbott SARS-CoV-2 IgG II Quant). The linear range of the assay is between 21 and 40,000 arbitrary units per milliliter (AU/mL) and the lower limit of detection (LoD) is 6.8 AU/mL. The clinical specificity is estimated at 99.55% (95% confidence interval (CI), 99.15–99.76%) and the clinical sensitivity at 98.81% (95% CI 93.56–99.94%) in the samples collected ≥15 days following a positive PCR at a cut-off value of 50 AU/mL, according to the manufacturer. Anti-SARS-CoV-2 RBD IgG assays have shown an excellent correlation with neutralizing antibodies [9,10].

Additionally, samples were tested for IgG antibodies directed against the SARS-CoV-2 nucleocapsid (N) protein, indicative of a previous infection, utilizing the Abbott SARS-CoV-2 IgG kit on an Architect i2000SR analyzer (Abbot Diagnostics, Green Oaks, IL, USA) according to the manufacturer’s instructions. An index [sample/calibrator (S/C)] with a positive cut-off of ≥1.4 was used [11]. Patients testing positive for anti-N abs were excluded from further analysis.

The study was approved by the ethics committee of the Onassis Cardiac Surgery Center.

### Statistical Analysis

Median values, 25th and 75th percentiles were used to describe anti-RBD levels. All samples below LoD 6.8 AU/mL were assigned the value 6.8 AU/mL. We compared levels among transplant organs and vaccine type using the non-parametric Mann–Whitney U test or Kruskal–Wallis test and chi-square test, respectively. The Poisson regression model with the robust sandwich standard error was used to identify factors associated with immune response. We conducted all statistical analyzes using Stata 13.1.

## 3. Results

A total of 455 SOT recipients were recruited. The majority of them, 372 out of 455 (81.8%) were kidney transplant recipients who were actively followed in the Nephrology and Renal Transplantation Clinic at Laiko Hospital in Athens. Of the remaining patients, all liver transplant recipients (*n* = 14) were followed in the Hepatology Unit of the same hospital and all heart (*n* = 46) and lung (*n* = 22) recipients at the Onassis Cardiac Surgery Center of Athens (Table 1).

There was a male predominance (65.1%). The mean (SD) age was 54.5 (13.7) years, and the median (25th, 75th) time from transplantation was 8.8 (3.6, 15.5) years. On study inclusion, approximately half of the patients (45.7%) were transplanted at an interval of 1–9 years, 49.2% for more than 9 years and only 23 recipients (5.1%) were transplanted for less than one year ago (Table 1).

More than two-thirds of patients (67.3%) had received the BNT162b2 and the remaining (32.7%) had the mRNA1273 vaccine. Almost all of the mRNA1273 vaccine recipients (145 out of 149) were kidney transplants (Table 2).

The vast majority of SOT recipients (95.9%) were on a calcineurin inhibitor (CNI)-based immunosuppressive regimen, mostly tacrolimus (72%), and less frequently cyclosporine (24.8%) in combination with an antimetabolite, mycophenolate acid in 81% and azathioprine in only 1.5% of patients. The mammalian target of rapamycin (mTOR) inhibitor everolimus was used less frequently in 14.9% of the total cohort. In general, our study population comprised stable SOT recipients, having been transplanted for a long time and receiving maintenance immunosuppression on the lowest edge, according to the individual immunological risk. Mean (±SD) trough levels of tacrolimus, cyclosporine, and everolimus were 6.0 (±1.3) ng/mL, 177 (±77) ng/mL and 5.2 (±1.2) ng/mL, respectively.

Kidney function was well-preserved with a mean (±SD) creatinine level of 1.5 (±0.6) mg/dl and a mean eGFR of 54.1 (±18.7) mL/min (Table 1).

### 3.1. Humoral Response

Anti-nucleocapsid, anti-(N) IgG antibodies were measured in 351 out of 455 patients (77.1%), and only six of them tested positive (1.7%) with median (25th, 75th) antibody levels: 368 (57, 1188) AU/mL. Notably, all of them had a positive history of previous COVID-19 infection, according to the questionnaire, and were subsequently excluded from further analysis (Table 1).

Only 39% (175 out of 449) of fully vaccinated SOT recipients tested positive for anti-SARS-CοV-2 RBD-IgG antibodies, and their median (25th, 75th) antibody titer was also low: 13 (7, 249) AU/mL.

When antibody titers were assessed according to the type of transplanted organ, antibody positivity was detected in 32.2% of kidney, 72.3% of heart, 77.3% of lung and 42.9% of liver transplant recipients, respectively.

**Figure 1:**
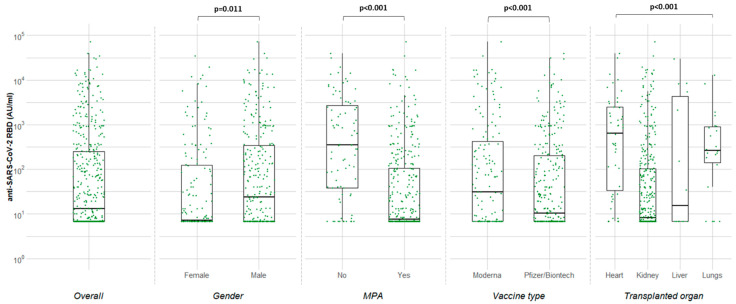
Median concentrations of anti-SARS-CoV-2 RBD (AU/mL) in vaccinated transplant recipient 15–45 days after the 2nd dose of BNT162b2 or mRNA-1273.

Finally, univariable, and multivariable analyzes were performed to identify factors associated with immune response. From the covariates assessed (adjusted multivariable RR (95% CI)), vaccine type (Moderna vs Pfizer/BioNTech) (1.96 (1.47–2.61)), younger age at transplantation (0.99 (0.98–1.00)), male gender (0.72 (0.5–0.93)), antimetabolite (1.91 (1.48–2.45))-and steroid-free IS and type of transplanted organ (heart (1.98 (1.39–2.82)) and lung (3.33 (2.42–4.57)) vs. kidney) were the factors independently associated with better immunogenicity (Table 3) (Figure 1).

### 3.2. Comparison of Response Rate between the Two mRNA Vaccines

We found a significant difference in favor of the mRNA1273 versus BNT162b2 vaccine in terms of antibody positivity (46.9% vs. 35.1%, *p* < 0.001), as well as median (25th, 75th) antibody levels: 31 (7, 486) AU/mL compared to 10 (7, 208) AU/mL, *p* = 0.016, respectively.

### 3.3. Adverse Events following Vaccination

Our study confirms existing evidence about the excellent safety of both mRNA vaccines in SOT recipients. We did not observe any serious adverse events immediately post-vaccination. The frequency of mild adverse effects was similar after the first and second dose in 69.5% and 62.9% of patients, respectively. The most common side effect was local pain at the injection site in 63.1% and 53.2% of patients after the first and the second dose, respectively. The second more frequent adverse effect was fatigue in 14.1% and 15.9% of patients, followed by headache (8.2% and 7.5%), myalgia (8.1% and 6.8%) and less commonly, fever (2.6% and 3.8%). Two months post-vaccination, almost all SOT recipients have completed at least one follow-up visit and we did not record any biopsy-proven acute rejection or otherwise unexplained organ dysfunction. Levels of SARS-CoV-2 RBD were not associated with self-reported adverse events.

## 4. Discussion

The present study is, to the best of our knowledge, the largest study investigating antibody response rates and the safety of the two mRNA SARS-CοV-2 vaccines in SOT recipients [12,13]. Most importantly, we compared the immunogenicity of the two available mRNA vaccines.

We found low response rates at 39% and low antibody titers among our SOT patients after the second vaccine dose, similarly to the response rate of 35% described in a recently published systematic review including a total of 1.744 SOT recipients [3].

In our cohort, response rates according to the type of transplanted organ were 32.2% in kidney, 72.3% in heart, 77.3% in lung and 42.9% in liver transplant recipients. Peled et al. have reported a low response rate of 18% in a cohort of 77 heart recipients; all had received two doses of the BNT162b2 vaccine [14]. Low response rates of 25% have been found in a study including 73 lung recipients who had received two doses of both mRNA vaccines [15], while response rates of 47.5% have been recorded in 80 liver transplant recipients, all who had been vaccinated with two doses of the BNT162b2 [16] vaccine. In a large series including 367 SOT recipients, Marion et al. have reported an overall response rate of 34% after at least one dose of vaccine; response rates assessed by transplanted organs were 33% in kidney, 12% in thoracic organ and 50% in liver recipients, respectively [17].

A possible explanation for the higher response rates among liver transplant recipients is the lower total amount of maintenance immunosuppression compared to the other SOT recipients. In the liver transplant cohort published by Rabinowich, the majority, 62.5% of patients were indeed on double and only 21.2% on triple immunosuppression [16]. In contrast, lung recipients are generally maintained at higher immunosuppression, with kidney and heart recipients being on an intermediate net level of immunosuppression between liver and lung recipients. Our response rates in kidney and liver transplant recipients are in accordance with the recently published literature [16,17]. The surprisingly high response rates in our heart recipients may be explained by the most frequently mTORi-based IS regimen (51% of heart recipients were on everolimus vs. only 11% of the kidney transplant recipients and 15% in the total cohort) while a plausible explanation for our high response rates in lung recipients might be their younger age (44.1 ± 9.8 years) compared to all other SOT recipients groups (*p* < 0.001).

Undoubtedly, the intensity of immunosuppression at the time of vaccination correlates with poor immunogenicity; therefore it is recommended to delay vaccination for one to three months after transplantation and for three months after anti-rejection treatment since high-dose steroids as well as treatment with antithymocyte globulin (ATG) have been associated with profoundly blunted immune responses [18]. In most studies, factors that are constantly associated with reduced immunogenicity to vaccination are antimetabolites, a short time since transplantation, older age and impaired renal function [4,19]. In our study, besides the BNT162b2 vaccine, among the covariates assessed, antimetabolite- and steroid-containing immunosuppression, female gender as well as older age at transplantation were factors that negatively influenced immune response.

Overall, regardless of the type of transplanted organ, our cohort comprised of stable transplant recipients with a long time after transplantation, and with well-preserved renal function on low-dose maintenance immunosuppression. Approximately one-third of our patients were weaned-off steroids, while trough levels of CNIs and mTORis were maintained at the lower edge.

A head-to-head comparison of the two mRNA vaccines revealed a difference in favor of the mRNA1273 vaccine in terms of creating a more robust humoral immunity regarding response rates (46.9% vs. 35.1%) and antibody titers as well. This finding is of particular interest since very few studies have addressed this issue. Boyarsky et al. first reported a response rate of 69% with the mRNA1273 vs. 31% with the BNT162b2 vaccine after the first vaccine dose in a cohort of 435 SOT recipients [13]. Haidar et al. investigated the immunogenicity of the two mRNA vaccines in a large study including healthy controls and different groups of immunocompromised patients. They found no difference in response rates between the two vaccines [7]. Interestingly, in a study in lung recipients, Narasimhan et al. found a trend towards a higher response rate (36% vs. 19%) in the mRNA1273 group, but the sample size was too small to reach statistical significance [15].

Regarding safety, current data on adverse events after vaccination with the mRNA vaccines in SOT recipients are reassuring [4,20]. We recorded only mild reactions in about two-thirds of our patients.

As feared by many transplant physicians, breakthrough infection is a major issue in SOT recipients and correlates with low or undetectable antibody titers. Most disturbingly, from the few data available, in transplanted patients, COVID-19 infection after full vaccination, does not seem to run a more indolent course [8,21].

The weak immune response and the unpredictable disease course of breakthrough infection have already prompted some national health authorities, such as the French, to recommend a third vaccination dose in SOT recipients. First reports confirm safety and improved immunogenicity after administration of the booster dose. In 101 SOT recipients, who all exclusively received the BNT162b2 vaccine, Kamar et al. showed a raise in response rate from 40% after the second to 68% after administration of the third dose [21]. Similarly, Benotmane et al. reported a response rate of 49% after the third dose in 159 SOT recipients who did not seroconvert after the second dose. All patients had received three doses of the mRNA1273 vaccine [22].

In view of our findings of stronger immunogenicity of the mRNA1273 vaccine, it would be reasonable to consider a booster with this vaccine in SOT recipients, regardless of the initial vaccination scheme.

Strengths of our study are that it includes the largest cohort of homogenously treated and followed SOT recipients in whom mRNA vaccine immunogenicity has been investigated until today and the testing of antibodies to nucleocapsid protein, which enabled the distinction between previously infected and naïve-vaccinated patients. Novelties of our study are the head-to-head comparison of the two mRNA vaccines.

Limitations include the absence of a healthy control group, the temporary absence of data about immunogenicity and safety after the third vaccine dose in this cohort of SOT recipients, and the lack of information about correlations with neutralizing antibodies and cellular immunity.

## 5. Conclusions

Our results support current evidence about low immunogenicity after vaccination with both the mRNA SARS-CοV-2 vaccines, with a better performance of the mRNA1273 vaccine in SOT recipients. These findings, besides the continuous practice of safety measures and “cocooning” vaccination of their close relatives, strongly implicate reevaluation of the vaccination policy in this immunocompromised patient population.

## Figures and Tables

**Table 1:** Demographics and clinical characteristics of the study population.

	N = 455
**Gender**, *n* (%)	
Male	296 (65.1)
Female	159 (34.9)
**Age** (years)	
mean (SD) ^1^	54.5 (13.7)
median (25th, 75th)	55.8 (44.8, 66.5)
**Country of birth**, *n* (%)	
Greece	407 (89.5)
Other	48 (10.5)
**Age at transplant** (years)	
mean (S.D.) ^1^	44.0 (13.8)
median (25th, 75th)	44.9 (34.9, 54.4)
**Time period between transplantation and 2nd dose** (years), median (25th, 75th)	8.8 (3.6, 15.5)
**Time period between transplantation and 2nd dose** (years), *n* (%)	
<1	23 (5.1)
1–9	208 (45.7)
>9	224 (49.2)
**Organ transplanted**, *n* (%)	
Kidney	372 (81.8)
Heart	46 (10.1)
Lung	22 (4.8)
Liver	14 (3.1)
Kidney/Heart	1 (0.2)
**Kidney function**	
**Creatinine** (μmol/L)	
mean (SD) ^1^	1.5 (0.6)
median (25th, 75th)	1.4 (1.1, 1.7)
**Estimated glomerular filtration rate (eGFR)** (ml/min)	
mean (SD) ^1^	54.1 (18.7)
median (25th, 75th)	54 (41, 64)
**Immunosuppression**	
**TAC**, *n* (%)	
Yes	327 (72.0)
No	127 (28.0)
**TAC levels** (ng/mL)	
mean (SD) ^1^	6.0 (1.3)
median (25th, 75th)	6.0 (5.3, 6.7)
**MPA**, *n* (%)	
Yes	359 (81.0)
No	84 (19.0)
**MP**, *n* (%)	
Yes	286 (64.7)
No	156 (35.3)
**CsA**, *n* (%)	
Yes	110 (24.8)
No	333 (75.2)
**CsA levels** (ng/mL)	
mean (SD) ^1^	177 (77)
median (25th, 75th)	165 (120, 225)
**Ever**, *n* (%)	
Yes	66 (14.9)
No	378 (85.1)
**EVER levels** (ng/mL)	
mean (SD) ^1^	5.2 (1.2)
median (25th, 75th)	5.3 (4.5, 6.0)
**Other**, *n* (%)	
Yes	7 (1.5)
No	449 (98.5)
**CNI = (TAC + CsA)**, *n* (%)	
Yes	425 (95.9)
No	18 (4.1)
**CNI + MPA + MP**, *n* (%)	
Yes	426 (96.4)
No	16 (3.6)
**CNI + MPA − MP**, *n* (%)	
Yes	2 (0.5)
No	440 (99.5)
**Transplant rejection** ^2^, *n* (%)	
Yes	3 (0.7)
No	451 (99.3)
**Anti-N**, *n* (%)	
Positive (≥1.4 AU/mL)	6 (1.7)
Negative (<1.4 AU/mL)	345 (98.3)

^1^ Standard deviation. ^2^ Last 3 months. Abbreviations: CNI, Calcineurin inhibitors; TAC, tacrolimus; CsA, Cyclosporine A; MPA, mycophenolic acid; MP, methylprednisolone; EVER, Everolimus.

**Table 2:** Vaccine type and vaccination status of the study population.

	N = 455
**Vaccine Type**, *n* (%)	
Pfizer–BioNTech	306 (67.3)
Moderna	149 (32.7)
**Side effects after the first dose of COVID-19 vaccine**, *n* (%)	
Yes	315 (69.5)
No	138 (30.5)
**Side effects after the first dose of COVID-19 vaccine** ^1^, *n* (%)	
Pain on the arm	285 (63.1)
Headache	37 (8.2)
Tiredness	64 (14.1)
Muscle pain	37 (8.1)
Fever	12 (2.6)
Skin rash	1 (0.2)
Other	22 (4.8)
**Side effects after the second dose of COVID-19 vaccine**, *n* (%)	
Yes	285 (62.9)
No	168 (37.1)
**Side effects after the second dose of COVID-19 vaccine** ^1^, *n* (%)	
Pain on the arm	241 (53.2)
Headache	34 (7.5)
Tiredness	72 (15.9)
Muscle pain	31 (6.8)
Fever	17 (3.8)
Skin rash	1 (0.2)
Other	28 (6.2)

^1^ Some participants had more than one symptom.

**Table 3:** Factors associated with the incidence of positive anti-RBD among solid organ transplant recipients in Greece, N = 449 ^1^.

Variable	Univariable RR (95% CI)	*p*-Value	Adjusted Multivariable RR (95% CI)	*p*-Value
**Age** (years)	0.99 (0.98–1.00)	0.002		
	0.98 (0.98–0.99)	<0.001	0.99 (0.98–1.00)	0.005
**Time from transplantation up to 2nd dose of vaccination** (years)	1.01 (1.00–1.02)	0.194		
**Gender**		0.007		0.011
Male	Ref.		Ref.	
Female	0.69 (0.53–0.90)		0.72 (0.57–0.93)	
**Vaccine**		0.013		<0.001
BNT162b2	Ref.		Ref.	
mRNA 1273	1.34 (1.06–1.68)		1.96 (1.47–2.61)	
**CNI = (TAC + CsA)**		0.999		
Yes	Ref.			
No	1.00 (0.55–1.81)			
**MPA**		<0.001		<0.001
Yes	Ref.		Ref.	
No	2.21 (1.79–2.72)		1.91 (1.48–2.45)	
**MP**		0.007		
Yes	Ref.			
No	1.38 (1.09–1.74)			
**EVER**		<0.001		
Yes	Ref.			
No	0.58 (0.46–0.73)			
**CNI + MPA + MP**		0.902		
Yes	Ref.			
No	1.04 (0.55–1.98)			
**CNI + MPA − MP**		0.725		
Yes	Ref.			
No	0.78 (0.19–3.13)			
**Organ transplanted**				
Kidney	Ref.		Ref.	
Heart	2.24 (1.78–2.83)	<0.001	1.98 (1.39–2.82)	<0.001
Lungs	2.40 (1.83–3.14)	<0.001	3.33 (2.42–4.57)	<0.001
Liver	1.33 (0.71–2.48)	0.371	1.57 (0.55–4.51)	0.402

^1^ Six participants with positive anti-nucleocapsid were excluded. Abbreviations: RR, risk ratio; CI, confidence interval; CNI, calcineurin inhibitors; TAC, tacrolimus; CsA, cyclosporine A; MPA, mycophenolic acid; MP, methylprednisolone; EVER, everolimus; anti-RBD antibodies against the receptor-binding domain.

## Data Availability

The data presented in this study are available on request from the corresponding author.
